# Association between serum IgM and all-cause mortality risk in Chinese centenarians: a prospective cohort study

**DOI:** 10.1186/s12979-024-00475-8

**Published:** 2024-10-16

**Authors:** Weiguang Zhang, Yuting Duan, Zhe Li, Yue Niu, Bin Wang, Zhe Feng, Ding Sun, Hao Li, Zehao Zhang, Zeyu Qu, Miao Liu, Hongyan Hu, Qiao Zhu, Yujian Chen, Chaoxue Ning, Shihui Fu, Shanshan Yang, Shengshu Wang, Yali Zhao, Yao He, Xiangmei Chen, Yizhi Chen

**Affiliations:** 1 Department of Nephrology, Hainan Hospital of Chinese PLA General Hospital, Academician Chen Xiangmei of Hainan Province Kidney Diseases Research Team Innovation Center, Sanya, 572013 China; 2 Senior Department of Nephrology, National Key Laboratory of Kidney Diseases, Beijing Key Laboratory of Kidney Diseases Research, the First Medical Center of Chinese PLA General Hospital, Chinese PLA Institute of Nephrology, National Clinical Research Center for Kidney Diseases, Beijing, 100853 China; 3 Department of Geriatric Medicine, The Affiliated Hospital of Qingdao University, Qingdao, 266000 China; 4 Luoyang Key Laboratory of Clinical Multiomics and Translational Medicine, Henan Key Laboratory of Rare Diseases, Endocrinology and Metabolism Center, The First Affiliated Hospital, College of Clinical Medicine of Henan, University of Science and Technology, Luoyang, 471003 China; 5 Institute of Geriatrics, Beijing Key Laboratory of Aging and Geriatrics, National Clinical Research Center for Geriatric Diseases, Second Medical Center, State Key Laboratory of Kidney Diseases, Chinese PLA General Hospital, Beijing, 100853 China; 6 Department of Laboratory Medicine, Hainan Hospital of Chinese PLA General Hospital, Sanya, 572013 China; 7 Central Laboratory, Hainan Hospital of Chinese PLA General Hospital, Sanya, 572013 China; 8 Department of Cardiology, Hainan Hospital of Chinese PLA General Hospital, Sanya, 572013 China; 9 Department of Disease Prevention and Control, First Medical Center, Chinese PLA General Hospital, Beijing, 100853 China; 10 The Second School of Clinical Medicine, Southern Medical University, Guangzhou, 510515 China; 11 Sanya Nephrology Medical Quality Control Center, Sanya, 572013 China

**Keywords:** Centenarians, Immunoglobulin M, Longevity, Risk of death, Immunity, Aging

## Abstract

**Background:**

We investigated the associations between IgM, IgG, IgA, and IgE levels and all-cause mortality risk in Chinese centenarians.

**Methods:**

All participants were from the China Hainan Centenarian Cohort Study. Eligible participants were divided into quartiles based on their IgM, IgG, IgA, and IgE levels. We used restricted cubic spline analyses, Cox regression analyses, and Kaplan–Meier survival curves to analyze associations between IgM, IgG, IgA, and IgE and all-cause mortality risk.

**Results:**

A total of 906 centenarian participants were included in this study (81.2% female; median age, 102 years). During a median follow-up of 30.1 months, 838 (92.5%) participants died. Restricted cubic spline analysis revealed a nonlinear relationship (“L” type) between serum IgM level and all-cause mortality. Compared with the higher three quartiles of serum IgM level, the lowest quartile was associated with a higher risk of death (Q1 versus Q2-Q4: HR, 1.365; 95% CI, 1.166–1.598; *P* < 0.001). Among individuals for whom IgM < 0.708 g/L (Q1), the risk of all-cause mortality was 36.5% higher. Kaplan–Meier analyses showed that centenarians with lower serum IgM levels had significantly shorter median survival time (Q1 versus Q2-Q4: 26 months versus 32 months, log-rank *P* = 0.001).

**Conclusion:**

Serum IgM levels in centenarians significantly correlated with the risk of death, suggesting that they are suitable for predicting the overall risk of death in centenarians and can be used as an independent predictor of death.

## Background

In general, immune function deteriorates with age [1]; however, there is a hypothesis that extreme survivors, such as centenarians, benefit from well-preserved, effective immune function and defense mechanisms [2]. Centenarians are considered the best examples of healthy human aging because they have not succumbed to major age-related diseases and reached the limit of human life, making them a good model for the study of healthy aging [3, 4]. With the pandemic of infectious diseases, the relationship between immunity and aging has received unprecedented attention in recent years, thereby highlighting the urgent need to combat immune aging and improve the immune function and resilience of older adults. When studying the immune function of centenarians, it is helpful to explore the key factors that may prolong the healthy lifespan of individuals and promote healthy aging.

Maintaining physiological homeostasis depends on a well-balanced immune system. B lymphocytes play a crucial role in humoral immunity by producing antibodies that recognize almost unlimited numbers of antigens. Some studies suggest that the number of B cells should be regarded as a sign of successful or unsuccessful aging [5]. Immunoglobulins or antibodies, proteins secreted by B lymphocytes, circulate in the body, where they mark, destroy, and/or neutralize bacteria, viruses, and other harmful or foreign substances (antigens) [6]. Immunoglobulins can be divided into five subtypes: IgM, IgD, IgG, IgA, and IgE, each of which has a series of different immune functions. In recent years, these immunoglobulins have been considered as novel biomarkers for chronic inflammation of the respiratory system [7], cardiovascular disease [8], liver disease [9, 10], and diabetes [11]. At the same time, studies have found that the level of immunoglobulin increases with age in the general population [12–14], and the higher the concentration, the greater the risk of death [15]. However, the number of articles related to the immune status of centenarians has been limited and the reported sample sizes have been small. The relationships between IgM, IgG, IgA, and IgE levels and all-cause mortality of centenarians are still unclear.

We conducted a study on a large cohort of centenarians with a median follow-up time of 30 months. The purpose of this study was to comprehensively evaluate the impact of circulating immunoglobulin (IgM, IgG, IgA, and IgE) levels on the risk of death in centenarians.

## Methods

### Study design and population

This study used data from the China Hainan Centenarian Cohort Study (CHCCS), a nationally representative prospective cohort study of centenarians conducted in Hainan Province between July 2014 and December 2016. A total of 1,002 centenarians participated in this study, and the baseline data have been reported in previous studies [16]. For each case, the date and cause of death were checked via the National Cause of Death Registration and Reporting Information System of the Chinese Center for Disease Control and Prevention, verified by the local civil affairs department, and confirmed by telephone survey of the family members, to ensure the accuracy of the follow-up results. The Hainan Provincial Civil Affairs Bureau, which is responsible for monthly pensions for individuals aged 80 and above, verifies at least monthly that the centenarians are alive. The study was approved by the Ethics Committee of the Hainan Hospital of Chinese People’s Liberation Army (PLA) General Hospital (No. 301HNLL-2016-01), and was conducted in accordance with the Declaration of Helsinki and its subsequent revisions.

### Covariates

All centenarians underwent a comprehensive visit and completed a questionnaire survey with the multidisciplinary clinical team of Hainan Hospital of the PLA General Hospital. Demographic information—such as age; sex; ethnicity; marital status; education; smoking history; alcohol history; status regarding diabetes mellitus (DM), hypertension, and coronary heart disease (CHD); weight, and height—was collected. BMI was calculated by dividing the weight by the square of the height. Blood samples were drawn from participants by an experienced nurse and sent to the Laboratory Department of Hainan Hospital of the PLA General Hospital. We used the immune light-scattering turbidity method to measure serum levels of IgM, IgG, IgA, and IgE using a fully automated protein analyzer (BNII; Siemens AG, Munich, Germany).

### Statistical methods

Data were tested for normality and homogeneity of variance before statistical analysis was performed. Normally distributed data were described as mean ± SD. Independent samples t-test or ANOVA were used to analyze differences between groups. Asymmetrically distributed data were described as median and interquartile range M (QL, QU) and Mann-Whitney U test or Kruskal-Wallis test was used to analyze differences between groups. Categorical variables were described as numbers with percentages n (%) and compared withχ^2^test. IgE exhibited a highly skewed distribution. After performing a logarithmic transformation, we found that log10(IgE) (LnIgE) approximates a normal distribution.

Restricted cubic spline (RCS) analyses were performed to analyze the associations between all-cause mortality and IgM, IgG, IgA, and LnIgE levels as continuous variables in both unadjusted models and multivariate-adjusted models. Serum immunoglobulin levels were grouped according to interquartile range (IQR). For IgM, Q1: 0.168 ≤ IgM < 0.708 g/L, Q2: 0.708 ≤ IgM < 1.020 g/L, Q3: 1.020 ≤ IgM < 1.400 g/L, and Q4: 1.400 ≤ IgM ≤ 4.540 g/L. For IgG, Q1: 8.310 ≤ IgG < 13.600 g/L, Q2: 13.600 ≤ IgG < 15.700 g/L, Q3: 15.700 ≤ IgG < 18.100 g/L, and Q4: 18.100 ≤ IgG ≤ 31.100 g/L. For IgA, Q1: 0.890 ≤ IgA < 2.530 g/L, Q2: 2.530 ≤ IgA < 3.330 g/L, Q3: 3.330 ≤ IgA < 4.250 g/L, and Q4: 4.250 ≤ IgA ≤ 9.990 g/L. For LnIgE, Q1: 1.42 ≤ LnIgE < 4.40, Q2: 4.40 ≤ LnIgE < 5.61, Q3: 5.61 ≤ LnIgE < 6.70, and Q4:6.70 ≤ LnIgE ≤ 9.08. Prior to engaging in Cox regression analyses, we conducted tests for the proportional hazards assumption for IgM, IgG, IgA, and LnIgE, followed by both univariate and multivariate Cox regression analyses in this study: the former to estimate the hazard ratio (HR) and 95% CIs for mortality, and the latter to adjust for confounding factors. Multivariate model 1 was adjusted for age, sex, ethnicity, marital status, BMI, education, smoking status, alcohol status, and status for DM, hypertension, and CHD. On the basis of the model 1 correction variables, immune indexes were added to multivariate model 2: C3, C4, kappa, lambda, IgG, IgA, LnIgE, and IgM. The distribution of time to death is shown in Kaplan–Meier survival curves and compared using the log-rank test. Differences for which *P* < 0.05 were considered statistically significant. Data processing was performed in R language software (version 4.0.2).

## Results

### Baseline characteristics and follow-up

We excluded patients with monoclonal gammopathy and other hematological disorders, as well as centenarians with chronic infectious diseases and incomplete data (*N* = 96), resulting in a final study population of 906 centenarians. The study population was divided according to IgM quartiles. Of the 906 centenarians who were finally included, 81.2% were female (Table 1). During a median follow-up of 30.1 months (IQR: 14.8–53.2), 838 (92.5%) of the centenarians died. The median age was 102 years (IQR: 101–104). Nearly three-quarters (74.0%) had hypertension, and minority had DM (9.1%) or CHD (4.4%). Most had normal serum IgM levels (93.3%), whereas small proportions had levels above or below the normal value (3.0% and 3.8%, respectively). More than half of participants had normal serum IgG levels (51.8%), and the rest had higher than normal values (48.2%). Serum IgA levels were normal in most participants (69.3%), and higher than normal in the remaining participants (30.7%). Serum IgE was at a normal level in less than one-third of participants (28.3%), and most individuals had higher than normal levels (71.7%). No participants had serum IgG, IgA, or IgE levels that were lower than normal.

**Table 1:** Characteristics of the study population based on IgM quartiles at baseline

Variable	Overall	Q1[0.168,0.708)	Q2[0.708,1.020)	Q3[1.020,1.400)	Q4[1.40,4.540]	*P*
N	906	227	226	226	227	
Age, years	102.00 (101.00, 104.00)	102.00 (101.00, 104.00)	102.00 (101.00, 105.00)	102.00 (101.00, 104.00)	102.00 (101.00, 104.00)	0.713
Female, %	736 (81.20)	175 (77.10)	176 (77.90)	185 (81.90)	200 (88.10)	0.010
Follow-up time, months	30.10 (14.80, 53.22)	26.00 (12.85, 44.85)	30.95 (12.30, 55.68)	30.85 (16.42, 50.03)	32.90 (16.00, 58.45)	0.022
Death, %	838 (92.50)	219 (96.50)	208 (92.00)	211 (93.40)	200 (88.10)	0.008
Ethnicity						0.866
Han, %	804 (88.70)	203(89.40)	201(88.90)	202(89.40)	198(87.20)	
Other, %	102 (11.30)	24 (10.60)	25 (11.10)	24 (10.60)	29 (12.80)	
Marital status						0.847
Married, %	96(10.60)	27(11.90)	24(10.60)	21(9.30)	24(10.60)	
Separation/Divorce/Widowhood, %	810 (89.40)	200 (88.10)	202 (89.40)	205 (90.70)	203 (89.40)	
Education						0.024
No education, %	824 (90.90)	204 (89.90)	203 (89.80)	203 (89.80)	214 (94.30)	
Elementary school, %	63 (7.00)	12 (5.30)	19 (8.40)	20 (8.80)	12 (5.30)	
Junior high school and above, %	19 (2.10)	11 (4.80)	4 (1.80)	3 (1.30)	1 (0.40)	
Smoke						0.332
Never, %	806 (89.00)	202 (89.00)	206 (91.20)	193 (85.40)	205 (90.30)	
Past, %	68 (7.50)	18 (7.90)	14 (6.20)	19 (8.40)	17 (7.50)	
Now, %	32 (3.50)	7 (3.10)	6 (2.70)	14 (6.20)	5 (2.20)	
Drinking						0.392
Never, %	746 (82.30)	187 (82.40)	188 (83.20)	178 (78.80)	193 (85.00)	
Past, %	71 (7.80)	16 (7.00)	21 (9.30)	22 (9.70)	12 (5.30)	
Now, %	89 (9.80)	24 (10.60)	17 (7.50)	26 (11.50)	22 (9.70)	
Hypertension, %	670 (74.00)	164 (72.20)	173 (76.50)	163 (72.10)	170 (74.90)	0.653
Diabetes mellitus, %	82 (9.10)	24 (10.60)	22 (9.70)	21 (9.30)	15 (6.60)	0.488
Coronary heart disease, %	40 (4.40)	12 (5.30)	11 (4.90)	12 (5.30)	5 (2.20)	0.267
Body mass index, kg/m^2^	18.15 (16.22, 20.08)	18.59 (16.42, 20.11)	18.04 (16.35, 20.00)	17.90 (16.05, 20.10)	17.86 (16.04, 19.81)	0.427
IgM, g/L	1.02 (0.71, 1.40)	0.56 (0.46, 0.64)	0.87 (0.78, 0.94)	1.19 (1.10, 1.28)	1.72 (1.52, 1.94)	< 0.001
IgM Categories						< 0.001
Low group (< 0.4 g/L), %	34 (3.80)	34 (15.00)	0 (0.00)	0 (0.00)	0 (0.00)	
Normal group (0.4–2.3 g/L), %	845 (93.30)	193 (85.00)	226 (100.00)	226 (100.00)	200 (88.10)	
High group (> 2.3 g/L), %	27 (3.00)	0 (0.00)	0 (0.00)	0 (0.00)	27 (11.90)	
IgG, g/L	15.70 (13.60, 18.10)	15.20 (12.90, 17.75)	15.50 (13.63, 1775.00)	15.70 (13.60, 18.20)	16.40 (14.30, 18.60)	< 0.001
IgG Categories						0.059
Normal group (7–16 g/L), %	469(51.80)	131(57.70)	118(52.20)	118(52.20)	102(44.90)	
High group (> 16 g/L), %	437 (48.20)	96 (42.30)	108 (47.80)	108 (47.80)	125 (55.10)	
IgA, g/L	3.33 (2.53, 4.25)	3.36 (2.54, 4.17)	3.32 (2.62, 4.14)	3.33 (2.46, 4.29)	3.33 (2.55, 4.31)	0.995
IgA Categories						0.700
Normal group (0.7–4 g/L), %	628(69.30)	163(71.80)	158(69.90)	151(66.80)	156(68.70)	
High group (> 4 g/L), %	278 (30.70)	64 (28.20)	68 (30.10)	75 (33.20)	71 (31.30)	
IgE, IU/mL	273.00 (81.50, 814.75)	264.00 (72.50, 698.00)	308.50 (97.25, 973.75)	293.00 (95.75, 878.50)	198.00 (68.00, 703.50)	0.184
IgE Categories						0.242
Normal group (0-100 IU/mL), %	256(28.30)	69(30.40)	57(25.20)	57(25.20)	73(32.20)	
High group (> 100 IU/mL), %	650 (71.70)	158 (69.60)	169 (74.80)	169 (74.80)	154 (67.80)	
LnIgE	5.56 ± 1.64	5.41 ± 1.60	5.69 ± 1.68	5.66 ± 1.61	5.48 ± 1.64	0.177
C3, g/L	0.98 (0.86, 1.11)	0.99 (0.87, 1.12)	0.97 (0.85, 1.08)	0.97 (0.86, 1.10)	1.00 (0.86, 1.16)	0.308
C4, g/L	0.23 (0.19, 0.28)	0.23 (0.19, 0.29)	0.22 (0.18, 0.27)	0.23 (0.19, 0.29)	0.23 (0.18, 0.29)	0.178
Kappa, g/L	4.13 (3.54, 4.78)	4.01 (3.42, 4.67)	4.12 (3.54, 4.71)	4.14 (3.58, 4.73)	4.30 (3.65, 5.01)	0.011
Lambda, g/L	2.09 (1.78, 2.49)	2.04 (1.72, 2.38)	2.03 (1.78, 2.35)	2.09 (1.80, 2.52)	2.21 (1.86, 2.65)	< 0.001

### RCS analyses of serum IgM, IgG, IgA, and LnIgE levels

**Fig. 1:**
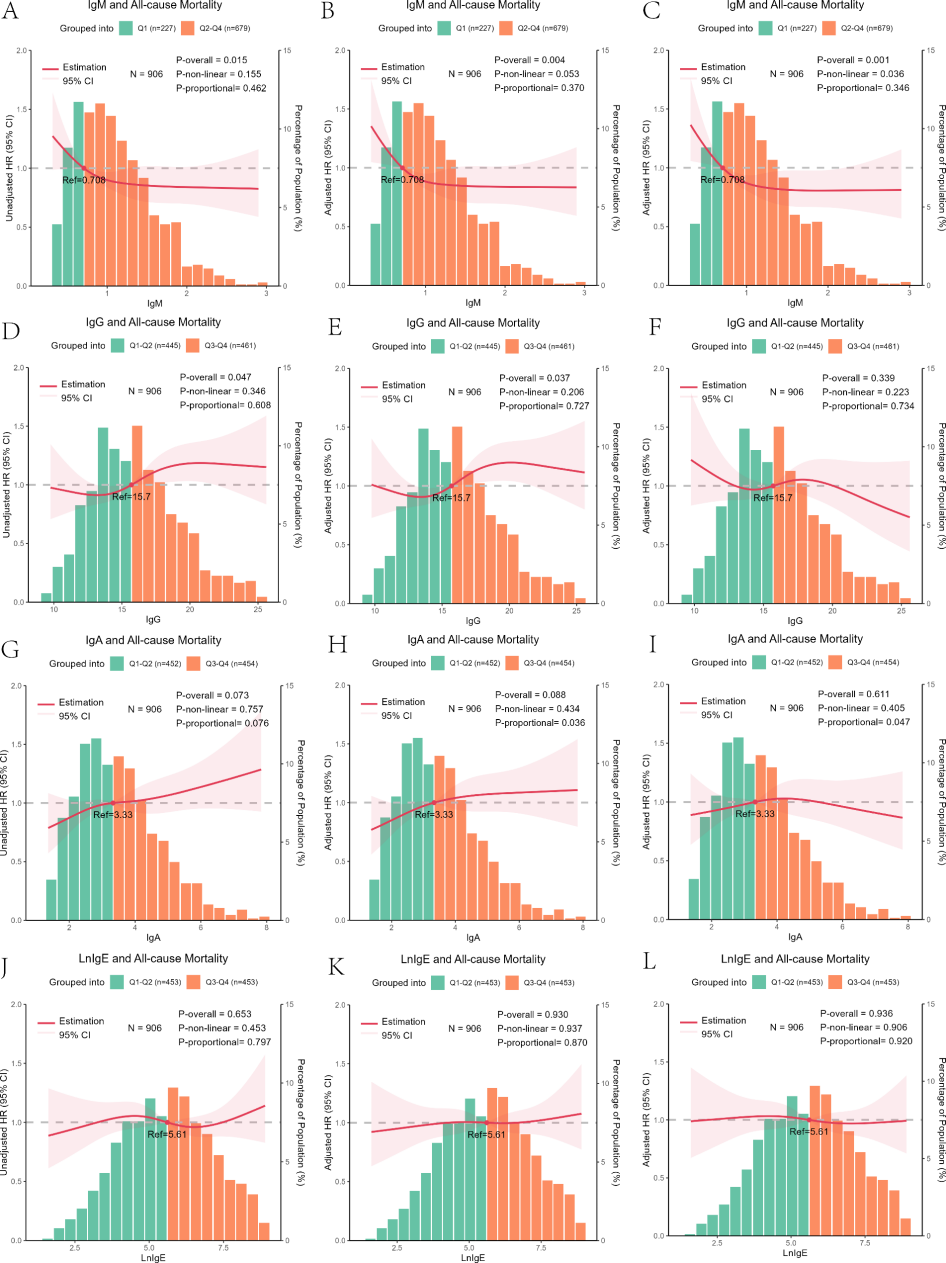
Restricted cubic spline models for the associations of IgM, IgG, IgA, and LnIgE with all-cause mortality risk. The solid red line indicates the HR and the shaded areas represent the 95% CI. The horizontal coordinates indicate IgM, IgG, IgA, and LnIgE levels, the left vertical coordinates indicate the HRs for all-cause mortality, and the right vertical coordinates indicate the percentages of centenarian participants with those IgM, IgG, IgA, and LnIgE levels. Univariate analysis (**A, D, G, J**). Multivariate analysis 1 adjusted for age, sex, ethnicity, marital status, BMI, education, smoking status, alcohol status, and status for diabetes mellitus, hypertension, and coronary heart disease (**B, E, H, K**). Multivariate analysis 2 adjusted for age; sex; ethnicity; marital status; BMI; education; smoking status; alcohol status; status for diabetes mellitus, hypertension, and coronary heart disease; and levels of C3, C4, kappa, lambda, IgM, IgG, IgA, and IgE (**C, F, I, L**). The reference cut-off value for IgM was the P25, 0.708 g/L. The relationship of IgM with all-cause mortality was linear in the unadjusted and multivariate analysis 1 (**A, B**), and non-linear in multivariate analysis 2 (**C**), restricted cubic spline models. The reference cut-off values were the respective medians: IgG, 15.7 g/L; IgA, 3.33 g/L; and LnIgE, 5.61. The relationship between IgG and all-cause mortality was linear in the unadjusted and multivariate analyses 1, and no significant differences in the multivariate analyses 2 (**D, E, F**). No significant differences were observed for IgA and LnIgE in the restricted cubic spline models (**G, H, I, J, K, L**)

Figure 1 shows the RCS analyses for serum IgM, IgG, IgA, and LnIgE. We conducted tests for the proportional hazards assumption for IgM, IgG, IgA, and LnIgE within the RCS model. The *p*-values for IgM, IgG, and LnIgE in both univariate and multivariate analyses, including Multivariate Analysis 1 and Multivariate Analysis 2, as well as for IgA in the univariate model, exceeded the threshold of 0.05. However, the *p*-values for IgA in both Multivariate Analysis 1 and Multivariate Analysis 2 were below 0.05. The RCS showed that, after adjusting for confounding factors, serum IgM levels were associated with the risk of death in an “L” pattern. With an increase in serum IgM level, the mortality risk for centenarians decreased before stabilizing (IgM: univariate analysis, *P* = 0.015, *P* nonlinear = 0.155; multivariate analysis 1, *P* = 0.004, *P* nonlinear = 0.053; and multivariate analysis 2, *P* = 0.001, *P* nonlinear = 0.036). Serum IgG levels were associated with all-cause mortality risk in univariate analysis and multivariate analysis 1 (*P* = 0.047 and *P* = 0.037, respectively), but not in multivariate analysis 2 (*P* = 0.339). Serum IgA levels were not significantly associated with mortality in any analyses (*P* = 0.073, *P* = 0.088, *P* = 0.611), nor were serum LnIgE levels (*P* = 0.653, *P* = 0.930, *P* = 0.936).

### Univariate and multivariate Cox analyses and Kaplan–Meier curves based on serum IgM, IgG, IgA, and LnIgE levels

Table 2 details the relationship between serum IgM, IgG, IgA, and LnIgE levels and the risk of all-cause mortality in centenarians. In the univariate model, the higher the serum IgM level, the lower the risk of death. Similar results were observed in both multivariate analyses. We used Q4, the group with the highest IQR, as the reference. In the univariate analysis, the risk of death was higher for Q1, Q2, and Q3 than for Q4, with the highest risk for Q1 (HR, 1.413; 95% CI, 1.166–1.712; *P* < 0.001). Similar results were observed in the multivariate analyses. In multivariate analysis 1, the risk of death was elevated for Q1, Q2, and Q3, with the highest risk for Q1 (HR, 1.445; 95% CI 1.189–1.758; *P* < 0.001). In multivariate analysis 2, Q1, Q2, and Q3 had elevated risks of death, with the highest risk for Q1 group (HR, 1.546; 95% CI 1.263–1.891; *P* < 0.001). When two groups were created based on the first quartile value, P25(Q1), similar results were found. The mortality rates for centenarians with serum IgM levels lower than P25 (< 0.708 g/L) were significantly higher than those with levels higher than P25 (≥ 0.708 g/L), and IgM level was significantly associated with mortality in univariate analysis (HR, 1.287; 95% CI, 1.103–1.502; *P* = 0.001), multivariate analysis 1 (HR, 1.319; 95% CI, 1.129–1.540; *P* < 0.001), and multivariate analysis 2 (HR, 1.365; 95% CI, 1.166–1.598; *P* < 0.001). That means for centenarians with an IgM level < 0.708 g/L, the all-cause mortality rate was 36.5% higher. The analyses of IgG, IgA, and LnIgE levels found that the risk of death was non-significantly higher among those with higher serum IgG and IgA levels, and there was no statistically significant difference among the serum LnIgE groups.

**Table 2:** Univariate and multivariate Cox analyses based on IgM, IgG, IgA, and LnIgE

Terms	Count	Univariate analysis		Multivariate -adjusted analysis 1		Multivariate-adjusted analysis 2	
IgM	906	HR	*P*	HR	*P*	HR	*P*
Grouped into quartiles							
Q1 [0.168,0.708)	227	1.413(1.166,1.712)	< 0.001	1.445(1.189,1.758)	< 0.001	1.546(1.263,1.891)	< 0.001
Q2 [0.708,1.020)	226	1.102(0.907,1.338)	0.329	1.098(0.909,1.349)	0.352	1.134(0.927,1.387)	0.222
Q3 [1.020,1.400)	226	1.204(0.992,1.461)	0.060	1.162(0.954,1.414)	0.136	1.221(0.992,1.480)	0.059
Q4 [1.400,4.540]	227	1		1		1	
*P* for trend			0.002		0.001		< 0.001
Q1	227	1.287(1.103,1.502)	0.001	1.319(1.129,1.540)	< 0.001	1.365(1.166,1.598)	< 0.001
Q2-Q4		1		1		1	
IgG	906						
Grouped into quartiles							
Q1 [8.310,13.600)	218	1		1		1	
Q2 [13.600,15.700)	227	1.032(0.850,1.253)	0.751	1.048(0.860,1.277)	0.641	0.999(0.812,1.230)	0.994
Q3 [15.700,18.100)	233	1.151(0.948,1.399)	0.156	1.164(0.956,1.416)	0.131	1.098(0.873,1.380)	0.425
Q4 [18.100,31.100]	228	1.334(1.099,1.619)	0.004	1.346(1.104,1.641)	0.003	1.141(0.842,1.548)	0.395
*P* for trend			0.002		0.002		0.311
IgA	906						
Grouped into quartiles							
Q1 [0.890,2.530)	223	1		1		1	
Q2 [2.530,3.330)	229	1.142(0.941,1.385)	0.180	1.144(0.941,1.390)	0.177	1.066(0.871,1.305)	0.535
Q3 [3.330,4.250)	227	1.257(1.036,1.526)	0.021	1.235(1.015,1.503)	0.035	1.141(0.925,1.406)	0.218
Q4 [4.250,9.990]	227	1.205(0.992,1.462)	0.060	1.233(1.013,1.500)	0.037	1.044(0.818,1.332)	0.730
*P* for trend			0.036		0.026		0.566
LnIgE	906						
Grouped into quartiles							
Q1 [1.420,4.400)	227	1		1		1	
Q2 [4.400,5.610)	226	1.080(0.891,1.309)	0.434	1.078(0.887,1.309)	0.453	1.018(0.836,1.240)	0.856
Q3 [5.610,6.700)	226	1.014(0.838,1.229)	0.883	1.059(0.870,1.290)	0.568	0.989(0.809,1.208)	0.910
Q4 [6.700,9.080]	227	0.977(0.806,1.183)	0.808	1.017(0.835,1.238)	0.867	0.915(0.749,1.119)	0.389
P for trend			0.667		0.906		0.361

Multivariate analysis 1 was adjusted for age, sex, ethnicity, marital status, BMI, education, smoking status, alcohol status, and status for diabetes mellitus, hypertension, and coronary heart disease

Multivariate analysis 2 was adjusted for age; sex; ethnicity; marital status; BMI; education; smoking status; alcohol status; status for diabetes mellitus, hypertension, and coronary heart disease; and levels of C3, C4, kappa, lambda, IgM, IgG, IgA, and LnIgE

**Fig. 2:**
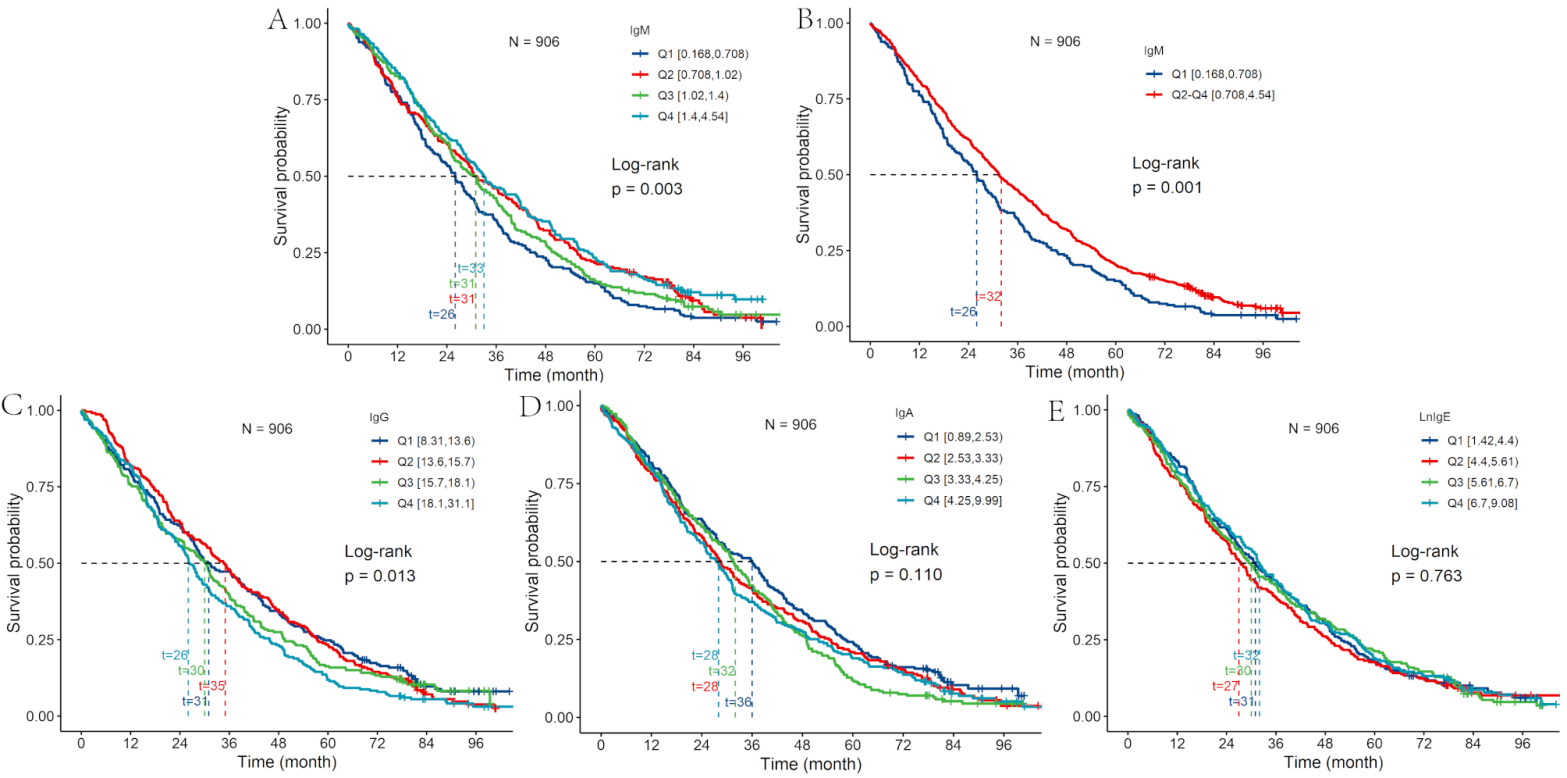
Kaplan–Meier survival curves based on IgM, IgG, IgA, and LnIgE, grouped into quartiles. The Kaplan–Meier survival curves revealed significant associations between higher IgM levels and increased survival time. The median survival time was significantly shorter for participants in the Q1 group than Q4 group on the serum IgM group (26 months versus 33 months, *P* = 0.003) (**A**). The median survival time was significantly shorter for participants in the Q1 group than Q2-Q4 group on the serum IgM group (26 months versus 32 months, *P* = 0.001) (**B**). Kaplan–Meier survival curves revealed associations between higher IgG levels and decreased survival time. The median survival time was shorter for individuals in the Q4 group than Q1 group on the serum IgG group (26 months versus 35 months, *P* = 0.013) (**C**). The median survival time did not significantly differ based on IgA and LnIgE levels (**D, E**)

Figure 2 shows the Kaplan–Meier curves and *P*-values of the differences in mortality based on the serum IgM, IgG, IgA, and LnIgE quartile. The survival curves revealed significant associations between higher IgM levels and longer survival time. The median survival time was significantly shorter for centenarians in the Q1 group than Q4 group on the serum IgM group (26 months versus 33 months; log-rank, *P* = 0.003). Grouped by the P25 IgM value, centenarians in the low group had a significantly shorter median survival time than those in the high group (26 months versus 32 months; log-rank, *P* = 0.001). The median survival time was shorter for centenarians in the Q1 group than Q4 group on the serum IgG group (26 months versus 35 months; log-rank, *P* = 0.013). The median survival time did not significantly differ for centenarians based on IgA and LnIgE, based on quartile.

## Discussion

This study is the first to investigate the associations between serum IgM, IgG, IgA, and IgE levels and the risk of all-cause mortality in centenarians in Hainan Province, China. Further, this is the first study of serum immunoglobulins and risk of death in centenarians with large-scale, long-term follow-up. Our study not only found that a lower serum IgM concentration was associated with mortality risk in centenarians, but also observed that this trend was nonlinear, such that a higher serum IgM level was associated with a lower mortality risk in centenarians before reaching a plateau. After adjusting for population characteristics, comorbidities, and other immune indicators in multivariate Cox regression analyses, the change in serum IgM concentration still significantly correlated with the risk of death, to a greater degree than IgG, IgA, and IgE level, suggesting that serum IgM is more suitable for predicting the overall risk of death in centenarians, and that it may be useful as an independent predictor of death.

IgM, part of the body’s adaptive humoral immune response against foreign pathogens, is the first type of antibody secreted by the adaptive immune system in response to foreign antigens [17]. It can be divided into natural IgM and antigen-induced IgM, which can be membrane-bound or secreted [18]. Natural IgM plays multiple roles in homeostasis, including promoting the clearance of apoptotic or dead cells, B cell survival signaling, and autoimmune disease prevention [19, 20]. IgM is increasingly recognized for its diverse roles in immune homeostasis, particularly in the context of infectious and non-infectious diseases. It serves as a frontline defense against a spectrum of pathogens, including protozoan parasites such as malaria, fungal infections, and is implicated in the pathogenesis of allergic conditions like asthma, as well as viral infections such as influenza A [21]. At mucosal sites, both natural IgM and antigen-induced IgM help shape a healthy microbiota [22]. Notably, IgM is thought to play a pivotal role in enhancing mucosal tolerance and, in conjunction with IgA, influencing the structure and function of the microbiota [21]. IgM has been observed to foster the proliferation of bacterial species that are instrumental for maintaining a balanced gut homeostasis, such as Firmicutes, including Bacillus cereus, Lachnospiraceae species, and Ruthenibacterium species [23, 24]. These bacteria are known to contribute to various metabolic processes in the gut, which are essential for the host’s immune response and overall health. In addition, natural IgM is associated with the recognition and clearance of premalignant cells because natural IgM antibodies are able to recognize carbohydrate-patterned autoantigens and rapidly activate complement [25]. Natural IgM antibodies can also inhibit microvesicle-driven coagulation and thrombosis [26]. These characteristics suggest that IgM may contribute to the ability of centenarians to avoid various fatal diseases.

Previous studies on IgM have mostly focused on autoimmune diseases [27], cancer [28, 29], and infectious diseases [30]. However, IgM can also protect blood vessels by neutralizing atherosclerotic antigens, and the level of natural IgM negatively correlates with atherosclerosis progression, carotid artery thickness, and the frequency of cardiovascular events [31–33]. In a study of 1,753 volunteers, Ramzi Y. Khamis and his colleagues discovered that, in patients with hypertension, the higher total serum IgM levels are independent predictors of freedom from cardiovascular events in general and from coronary heart disease in particular [34]. Our findings echo those of the Ramzi Y. Khamis study, suggesting that there is a negative correlation between serum IgM level and all-cause mortality in centenarians. The higher the serum IgM level, the lower the mortality risk, up to the point that the risk plateaus. Phillips AC et al. investigated a cohort of 4255 Vietnam-era, former US army personnel (the Vietnam Experience Study) who served during the Vietnam War and found that elevated levels of immunoglobulin were associated with an increased risk of all-cause mortality and deaths attributed to “other” causes, predominantly infectious diseases [15]. This finding contrasts with our results, which may be attributed to several factors. Firstly, the study cohort comprised exclusively male military personnel, whereas in our study, females constituted 81.2% of the population, potentially contributing to the observed discrepancies. Secondly, the age at baseline in the Phillips AC et al. study was 38.6 ± 2.68 years for decedents and 38.3 ± 2.51 years for survivors. Thirdly, the study did not exclude participants with infectious diseases, whereas our study rigorously excluded such individuals. Additionally, centenarians may possess unique immune regulatory mechanisms that contribute to their health and longevity. Florinda et al. studied the serum immunoglobulin levels of 166 subjects (20–106 years old) and found that IgG and IgA levels increased in an age-related manner, whereas IgE levels remained unchanged and serum IgD and IgM levels decreased with age; the age-related decline in IgM was only statistically significant in women, which may be related to the relatively small number of men in their sample (male: female = 49:117) [35]. However, in 2010, Colonna-Romano and colleagues conducted a comparative experiment of immunosenescence in the offspring of 29 centenarians and 25 age-matched controls. The experiment found that naïve B cells and the IgM ratio were higher in the offspring of centenarians compared with the control group [5]. Similarly, our study found that IgM level was associated with the mortality risk of centenarians in an “L” pattern. With a higher serum IgM level, the mortality risk of centenarians decreased before leveling off. At the same time, we found that 93.3% of centenarians’ serum IgM levels were at the normal level, which confirmed that IgM was associated with long life, likely owing to its ability to help centenarians resist disease, so IgM could be useful as an independent predictor of all-cause mortality in centenarians. To date, the relationship between IgM and all-cause mortality in centenarians has not been fully explored. Therefore, this study provides the first evidence for the relationship between serum IgM levels and all-cause mortality in centenarians.

IgG is the most abundant type of immunoglobulin in the human body, and IgG antibodies usually have high affinity, making them particularly effective at neutralizing secreted bacterial toxins and viral docking molecules used to enter cells [36]. IgA is secreted at mucosal surfaces (such as the mouth, nose, and gastrointestinal tract), where they are the first line of defense against surface infections and pathogen colonization [37]. Compared with other isotypes, IgE is present at very low concentrations in the body; it is related to responses to parasitic infections, such as those caused by helminths [38]. Interestingly, in our study, the percentages of centenarians with higher IgA, IgG, and IgE above the normal ranges were approximately 30%, 50%, and 70%, respectively, and there were no centenarians with IgG, IgA, and IgE below the normal range, which suggested that the immune function of the centenarians was normal or enhanced. This is similar to the findings of an earlier observational report, that the immune function of healthy centenarians is closer to that of young people than that of 70-year-old individuals [39]. In another study, the biological age of supercentenarians (> 110 years old) was estimated by an epigenetic clock, and their epigenetic age was found to be 8.6 years younger than their actual age [40]. Franceschi et al., based on their study of “healthy” centenarians (i.e., subjects in good mental and physical condition), found evidence of sustained remodeling of the immune system, rather than general deterioration [2, 41]. The function of the immune system can regulate the speed of aging to slow down the inevitable aging process, which plays a key role in most chronic diseases of the elderly. The literature on immunosenescence has mainly focused on T cells [42, 43], while the B cell compartment is also affected in older adults. Colonna et al. found that changes in B cell subsets in centenarian offspring may mark successful or unsuccessful aging, suggesting B cell count could be used as a biomarker of human lifespan, allowing evaluation of anti-aging treatment [5]. Proteomic analysis revealed that healthy centenarians exhibited greater B cell-mediated immune responses, including antibody production, compared with controls from the same geographical area who died prematurely [44]. Our results also support the relationship between immunoglobulins secreted by B lymphocytes and longevity, adding strong evidence for the study of healthy immune system aging, especially the relationship between healthy aging and immunoglobulins.

This study focused on the long-lived population in Hainan, China, which is one of the few long-lived island populations in the world, with a large sample of centenarians. On the one hand, the study of long-lived people is conducive to the discovery of indicators closely related to health and longevity. On the other hand, serum IgM is more suitable for predicting the overall mortality risk of centenarians, with the potential for use as an independent predictor of death. Circulating immune proteins are easy to obtain and widely used, and can be used as immune biomarkers to monitor the susceptibility of older individuals to aging-related diseases.

This study has the following advantages. It uses the scarce resource of centenarians as a model of successful aging, with a follow-up of approximately 30 months. This may be the first study on the relationship between serum IgM, IgG, IgA, and IgE levels and mortality risk in a large, prospective cohort of centenarians. Our study shows that serum IgM level can be used as an independent risk factor to predict the mortality risk of centenarians, and further exploration may reveal it to be a potential biomarker of a long, healthy lifespan. The study also has some shortcomings. First, the study population is mainly derived from the Chinese Han population and the oldest adult population, so our results may not be applicable to populations with a more diverse ethnic composition or younger individuals. Second, the association between the combined levels of IgM, IgG, IgA, and IgE and the risk of death was not analyzed; this will be further explored in the next study. Third, we only explored the relationship between levels of IgM, IgG, IgA, and IgE and the risk of all-cause death. Further research will be required to determine the association of immunoglobulin levels with specific causes of death. Fourthly, we did not detect circulating B cell subsets and immunoglobulin subclasses in the population, however, we plan to detect and analyze them in future studies.

## Conclusion

Our study confirms that IgM is independently associated with all-cause mortality in centenarians, and we expect IgM to become a novel marker for healthy longevity.

## Data Availability

The raw data supporting the conclusions of this article will be made available from the corresponding author upon request and without undue reservation.

## Abbreviations

DM: Diabetes mellitus
CHD: Coronary heart disease
HR: Hazard ratio
RCS: Restricted cubic spline
IQR: Interquartile range
